# Protein Delivery of an Artificial Transcription Factor Restores Widespread Ube3a Expression in an Angelman Syndrome Mouse Brain

**DOI:** 10.1038/mt.2015.236

**Published:** 2016-02-02

**Authors:** Barbara J Bailus, Benjamin Pyles, Michelle M McAlister, Henriette O'Geen, Sarah H Lockwood, Alexa N Adams, Jennifer Trang T Nguyen, Abigail Yu, Robert F Berman, David J Segal

**Affiliations:** 1 Genome Center, MIND Institute, and Department of Biochemistry and Molecular Medicine, University of California, Davis, California, USA; 2 Department of Neurological Surgery, University of California, Davis, California, USA

## Abstract

Angelman syndrome (AS) is a neurological genetic disorder caused by loss of expression of the maternal copy of *UBE3A* in the brain. Due to brain-specific genetic imprinting at this locus, the paternal *UBE3A* is silenced by a long antisense transcript. Inhibition of the antisense transcript could lead to unsilencing of paternal *UBE3A*, thus providing a therapeutic approach for AS. However, widespread delivery of gene regulators to the brain remains challenging. Here, we report an engineered zinc finger-based artificial transcription factor (ATF) that, when injected i.p. or s.c., crossed the blood–brain barrier and increased Ube3a expression in the brain of an adult mouse model of AS. The factor displayed widespread distribution throughout the brain. Immunohistochemistry of both the hippocampus and cerebellum revealed an increase in Ube3a upon treatment. An ATF containing an alternative DNA-binding domain did not activate Ube3a. We believe this to be the first report of an injectable engineered zinc finger protein that can cause widespread activation of an endogenous gene in the brain. These observations have important implications for the study and treatment of AS and other neurological disorders.

## Introduction

Angelman syndrome (AS) is a neurological disorder of genetic origin that affects ~1:15,000 births and is characterized by intellectual disabilities, lack of speech, ataxia, and seizures.^1,2^ The genetic cause of AS is loss of UBE3A (ubiquitin-protein ligase E6-AP) expression in the brain, usually due to a 4-Mb *de novo* deletion of the maternal 15q11–q13 region, but also caused by imprinting defects, paternal uniparental disomy, or point mutations in the maternal *UBE3A* allele.^3,4^ Due to brain-specific imprinting, the paternal allele is silenced, thus loss of the maternal allele causes UBE3A deficiency throughout the brain.^5,6^

**Figure 1:**
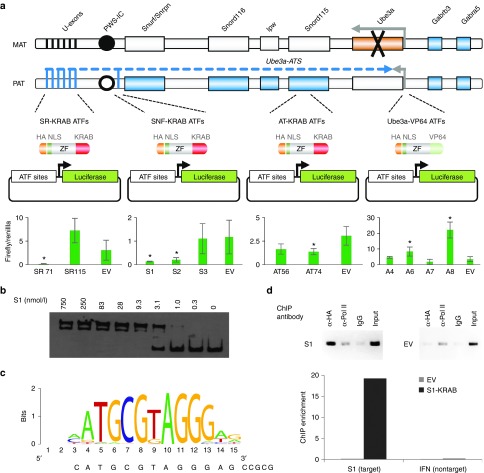
**ATF targeting strategy and binding results**. (**a**) Top: ATFs are shown in relationship to the genomic region on the mouse chromosome 7. Imprinting in this region results in genes with paternal-only, maternal-only, or silenced expression (active genes, filled; silenced genes, open). Genes outside this region are biallelically expressed. In the Angelman syndrome mouse model used in these studies, a targeted insertion with a stop codon replaces exon five (coding exon 2) of the maternal *Ube3a* (black X). U-exons, upstream exons of the *Ube3a-ATS*; PWS-IC, Prader–Willi syndrome imprinting control region either methylated (filled circle) or unmethylated (open circle). Bottom: Luciferase assays of 11 ATFs in HEK293T cells. Bars indicate the firefly luciferase signal, normalized to renilla luciferase control. Error bars indicate SD. *P < 0.01; two-tailed heteroscedastic *t*-test, representing three biological replicates. (**b**) Representative electromobility shift assay of S1 ZF array binding its target. (**c**) *In vitro* binding motif of the TAT-S1 ATF determined by Bind-n-Seq analysis. The sequence of the S1 target site is below. The motif represents a 56-fold enrichment over random background, *P* < 0.001, one-tailed Fisher's Exact Test. (**d**) ChIP–PCR data for the HA-tagged ATF S1 or EV at the S1 chromosomal target site in mouse Neuro2A cells. IgG serves as a negative control. The graph shows ChIP-enrichment relative to 0.1% chromatin input. ATF, artificial transcription factor; ChIP, chromatin immunoprecipitation; EV, empty vector; IFN, interferon; ZF, zinc finger.

A traditional gene therapy approach using adeno-associated virus and cDNA to restore *Ube3a* expression in the brain of a mouse model of AS resulted in a partial rescue of learning and memory defects but not motor coordination.^7^ These results may be explained by the limited distribution of viral vectors in the brain. Other approaches have attempted to reactivate (“unsilence”) the paternal *Ube3a* allele, with varied success for molecular and phenotypic rescue. Paternal silencing is caused by a long brain-specific RNA transcript that overlaps and is antisense to *Ube3a* (*Ube3a-ATS*; **Figure 1a**). The initiation, splicing, and function of this transcript are complex. The promoter of the *Snurf*/*Snrpn* locus is one start site for the *Ube3-ATS*, but numerous upstream start sites and exons create transcripts that, in mouse, can be over 1,000 kb.^8^ Inhibiting the extension of this transcript by insertion of a transcriptional termination sequence,^9^ topoisomerase inhibitor drugs,^10^ or antisense oligonucleotides^11^ results in partial unsilencing of paternal *Ube3a*, and some phenotypic rescue. These previous studies support that inhibition of *Ube3a-ATS* is a valid strategy to enhance paternal Ube3a expression.

In this study, we designed artificial transcription factors (ATFs) with the goal of increasing paternal expression of *Ube3a* in the brain of an adult mouse model of AS. ATFs are composed of a programmable DNA-binding domain, such as zinc fingers, TALEs or CRISPR/Cas systems, and an effector domain that can activate or repress transcription.^12,13^ Zinc finger-based ATFs have been evaluated in phase 2 clinical trials (NCT00476931), which demonstrated that zinc finger ATFs could be well tolerated in human subjects. The lack of similar data for TALE and CRISPR ATFs led us to choose zinc fingers for our initial studies. However, delivery of such ATFs throughout the brain has remained an unmet challenge. In particular, the blood–brain barrier created by the vascular endothelium in the central nervous system limits the method of delivery for most viral vectors, biologics, and drugs to i.c. or i.t. injection, which may be unfavorable for repeated treatments. Since ATFs only provide transient gene regulation they would need to be administered on a regular schedule to be effective for long-term gene regulation. In this study, we explored an alternative delivery approach utilizing an HIV TAT cell-penetrating peptide, which has been shown capable of delivering large proteins to the entire brain from a single i.p. injection.^14,15^ We observed that a purified, TAT-linked, zinc finger-based ATF repression protein targeted to the *Snurf*/*Snrpn* promoter was able to be injected i.p. or s.c., cross the blood–brain barrier, and activate endogenous Ube3a expression in multiple brain regions of a maternally *Ube3a*-deficient mouse model of AS.^16^ Such an approach could eventually be useful for the treatment of AS and other neurological conditions.

## Results

### Highly active zinc finger ATFs were identified by luciferase assay

In principle, activation of paternal *Ube3a* can be achieved either by super activation of the *Ube3a* promoter or by repression of the inhibitory *Ube3a-ATS* transcript. Eleven zinc finger arrays consisting of six zinc finger modules each were constructed to 18-bp target sites (**Supplementary Table S1**), including four to a region upstream of the *Ube3a* locus, three upstream of the *Snurf/Snrpn* locus (thought to be an initiation site of the *Ube3a-ATS*^17^), two to sites common in the approximately nine upstream initiation sites of the *Ube3a-ATS*,^8^ and two to a region between the *Snord115* cluster and *Ube3a* (**Figure 1a**, top). Activating factors contained a VP64 transcriptional activation domain^18^ and were assayed in HEK293T cells using a reporter plasmid in which luciferase was driven by the Ube3a promoter (considered to be a 1,000-bp region upstream of the transcriptional start site). Repressing factors contained a KRAB transcriptional repression domain^18^ and were assayed using luciferase reporter plasmids that harbored the target sites upstream of an SV40 promoter. The factor that provided the most robust activation was A8, and the two factors that produced the most potent repression regulatory effect (>fivefold repression, *P* < 0.015) were SR71 and S1 (**Figure 1a**, bottom, >fivefold regulation, *P* < 0.01).

### Creation of a purified fusion ATF protein for i.p. or s.c. injections

**Figure 2:**
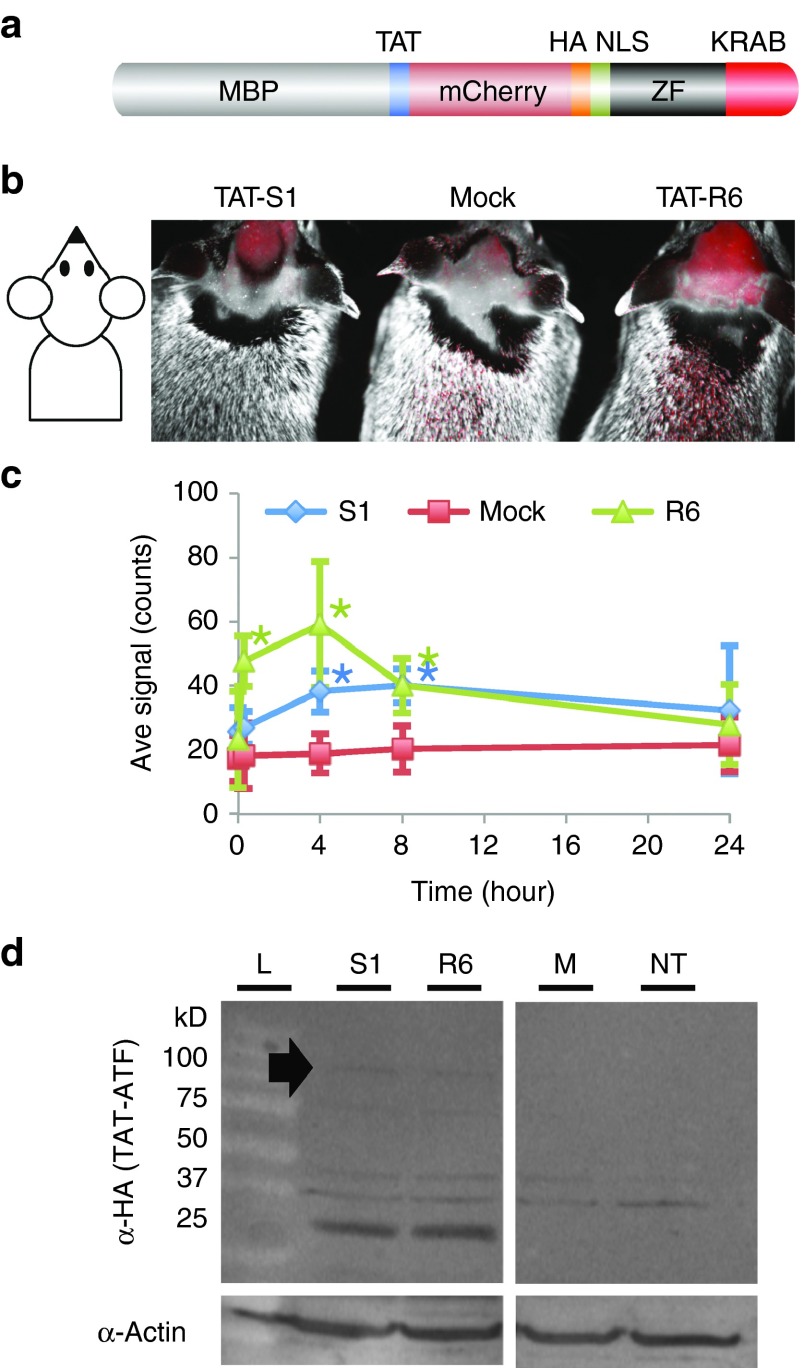
**Distribution of TAT-S1 and TAT-R6 in adult mouse brain**. (**a**) Structure of TAT-S1 and TAT-R6 ATF proteins. (**b**) mCherry fluorescence/ambient light merged image of live *Ube3a*-deficient mice 4 hours postinjection with TAT-S1, ATF injection buffer (Mock), or the negative control TAT-R6 (0.16–0.20 g/kg, i.p.). Fur was shaved to improve the fluorescent signal. (**c**) Kinetics of fluorescence in the C57BL/6 brains for TAT-S1 (green triangles), Mock (red squares), and TAT-R6 (blue diamonds). *P < 0.005 compared to Mock at that timepoint, two-tailed homoscedastic *t*-test, *n* = 5. (**d**) Western blot detecting the HA tag in brain nuclear lysate 4 hours postinjection. Filled arrow, 100-kD full-length protein band. Potential lower molecular weight breakdown products containing the HA tag are also visible. ATF, artificial transcription factor; M, mock injection; MBP, maltose-binding protein; NT, no treatment; S1, TAT-S1; R6, TAT-R6; ZF, zinc finger.

To investigate the potential of an injected ATF to regulate an endogenous gene in the brains of mice, we constructed fusion proteins for the most efficient regulators S1, SR71, and A8. These fusions contained an N-terminal maltose-binding protein for purification, a cell-penetrating peptide consisting of the 10-aa transduction domain of the HIV-transactivator protein (TAT, residues 48–57^19^), mCherry red fluorescent protein to aid in protein solubility and visualization, an HA epitope tag for detection, and an SV40 nuclear localization signal to ensure nuclear delivery. The TAT cell-penetrating peptide had been previously used to deliver proteins to the brains of mice following i.p. injection.^20^ The KRAB/VP64 domain was appended to the C-terminus of the zinc finger protein giving rise to TAT-S1, TAT-SR71, and TAT-A8 (**Figure 2a** and **Supplementary Figure S1**). Of these three constructs, only TAT-S1 was able to be purified in sufficient quantity (mg) for multiple mouse injection studies (data not shown). An electromobility shift assay (EMSA) demonstrated that S1 zinc finger array bound its target with an affinity of 5 ± 1.5 nmol/l (**Figure 1b**). The EMSA was repeated with the TAT-S1 and showed similar affinity of 8 ± 1.5 nmol/l (data not shown). The binding preference of the TAT-S1 was investigated using the *in vitro* Bind-n-seq assay, in which the protein was incubated with oligonucleotides representing all possible 21-bp binding regions, and the preferred targets were identified by next-generation sequencing.^21^ The observed binding motif indicated a high specificity for the intended target sequence (**Figure 1c**, 56-fold enrichment over background, *P* < 0.001).To evaluate S1 binding to the target locus *in vivo*, we performed chromatin immunoprecipitation followed by PCR in mouse Neuro2A cells. The S1 zinc finger array bound strongly to its intended chromosomal target site *in vivo*, while no binding was observed at an unrelated promoter (**Figure 1d**). Thus, the TAT-S1 showed substantial affinity and specificity for its target site.

### Injected TAT-ATFs distribute widely in mouse including the brain within 4–8 hours

**Figure 3:**
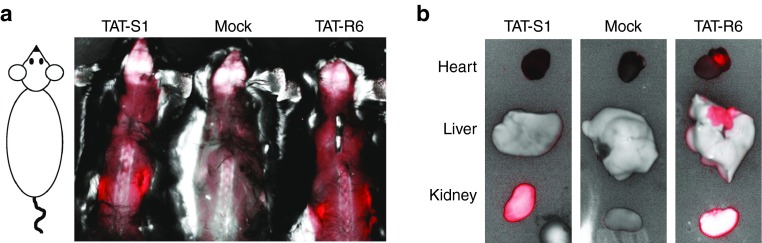
**Artificial transcription factor distribution in other organs**. (**a**) mCherry fluorescence/ambient light merged image of mice harvested 4 hours postinjection with TAT-S1, injection buffer (Mock), or TAT-R6 (0.16–0.20 g/kg, i.p.). The skin on the back was removed to improve the fluorescent signal. Intense signal can be seen in the kidneys. (**b**) Internal organs harvested after 4 hours. Note that the bright white kidney in the TAT-R6-injected sample indicates fluorescence in excess of the maximum setting.

To confirm that the upregulation of Ube3a was due specifically to TAT-S1, a negative control construct was made with zinc finger array R6 that was not designed to bind at the *Snurf/Snrpn* locus (chromosome 7). The closest match in the mouse genome contained two mismatches and was >30,000 bp from the nearest gene on chromosome 6. TAT-S1 and TAT-R6 differ by only 26 aa in their DNA-binding domains. The 922-aa TAT-ATFs (S1 and R6) were expressed in *Escherichia coli* using a cold temperature induction (see Materials and Methods) and purified (**Supplementary Figure S2**). Injection of adult wild-type C57BL/6 mice with purified TAT-S1 and TAT-R6 (160–200 mg/kg, i.p.) produced a significant peak of mCherry fluorescence in the brain within 4–8 hours (*P* < 0.005; **Figure 2b**,**c**). Full-length TAT-ATF protein could be detected by western blot analysis of brain nuclear lysates, in addition to potential lower molecular weight breakdown products (**Figure 2d**). TAT-ATFs also distributed to other parts of the body (**Figure 3**), although lysates of these organs did not show any detectable full length TAT-ATF (data not shown). The data suggest that TAT-ATFs injected i.p. can cross the blood–brain barrier and enter nuclei in the cells of the brain.

### Injected TAT-S1, but not TAT-R6, increases endogenous Ube3a expression in the brains of AS mice

**Figure 4:**
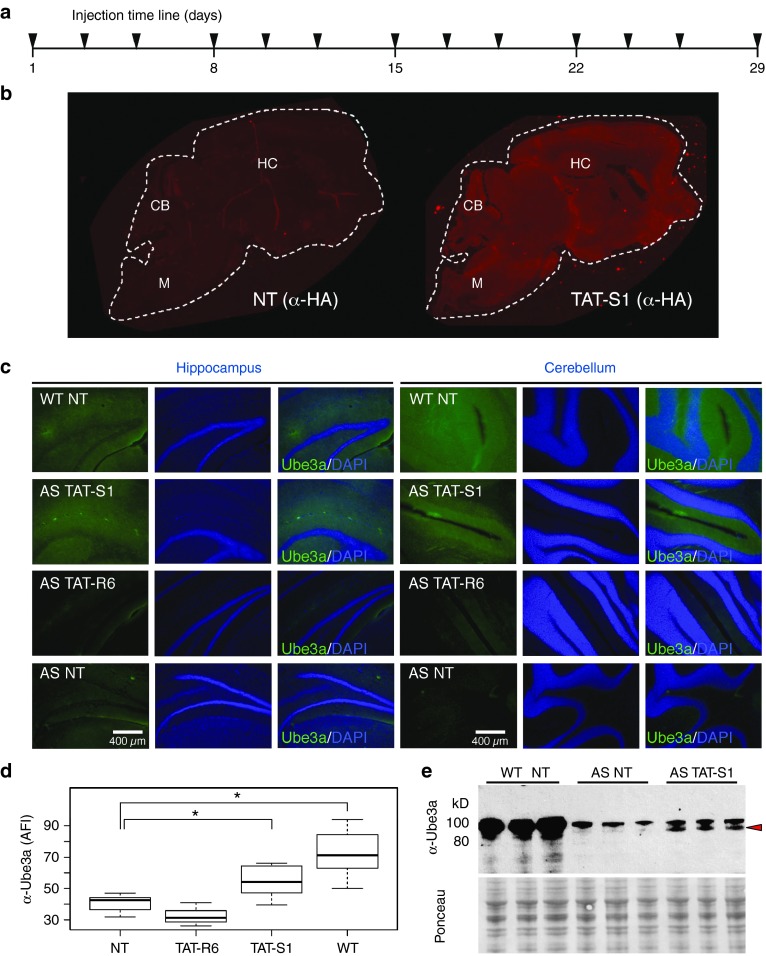
**Reactivation of Ube3a in a mouse model of AS by TAT-S1 but not TAT-R6**. (**a**) Artificial transcription factors were injected (160–200 mg/kg, s.c.) three times per week for 4 weeks, with the final inject 4 hours before harvest. (**b**) TAT-S1 distribution in a whole brain sagittal section (HA, 5 µm) from wild-type mice receiving NT or TAT-S1. White dashed line indicates outline of brain section. Images were not altered. (**c**) Imaging of Ube3a protein expression (green) or DAPI (blue) in brain slices of the hippocampus and cerebellum (Ube3a (Sigma E8655), 50 µm). Sections from NT wild-type and AS mice are shown as controls. A 10% linear brightness reduction was applied equally to the green channel of all images to reduce autofluorescence and clarify features. (**d**) Quantification of Ube3a from unaltered images of the same regions in different mice. One-way analysis of variance found significant difference between groups (*F*(3,18) = 15.5, *P* < 0.0001), *n* = 3–4 mice. *P < 0.01, *post hoc* Tukey–Kramer honest significant difference. (**e**) Western blot of Ube3a from brain cytosolic lysates of three different mice that received the indicated treatment (α-Ube3a, Sigma E8655). Red arrow indicates isoform that is specific to both the WT and TAT-S1-treated mice, but absent in the AS NT controls. Ponceau S staining of the membrane is shown as a loading control. AFI, average fluorescence intensity; AS, Angelman syndrome; CB, cerebellum; DAPI, 4′,6-diamidino-2-phenylindole; HC, hippocampus; M, medulla; NT, no treatment; WT, wild type.

To investigate if TAT-S1 could perform its gene regulatory function in the brains of mice, we used the Jiang *et al*.^16^ mouse model of AS that carried a transgene insertion in exon 5 (coding exon 2) at the 5′ end of the maternal *Ube3a* gene (AS mice, see **Figure 1a**). The insertion causes strong attenuation of Ube3a protein expression from the maternal allele. Adult mice (≥2 months) were used for these experiments since the *Ube3a-ATS* would be fully expressed from the paternal allele^22^ and would more accurately reflect an age of human diagnosis (3–4 years old), unlike genetic crosses or early postnatal interventions. Based on the anti-Ube3a-ATS studies described earlier,^9,10,11^ we expected our TAT-S1 to activate Ube3a protein expression from the paternal allele in this mouse model. However, given the half-life of our protein's signal in the brain is between 8 and 24 hours, and the KRAB effector domain is known to provide only transient transcriptional repression,^23^ we reasoned that multiple administrations are necessary to ensure sufficient presence of the ATF over time to alter gene expression patterns. The ATFs were injected (160–200 mg/kg, s.c.) three times per week for 4 weeks (**Figure 4a**). A final injection was given four hours before harvest to examine distribution of the ATF.

We observed no overt signs of toxicity during the 4-week treatment period, based on normal appearance and behaviors of the mice and no visual organ pathology upon dissection (data not shown). We observed widespread distribution of ATFs throughout all regions of the brain (**Figure 4b**). Ube3a protein was found to be significantly increased in AS mice treated with TAT-S1, but not TAT-R6, in both hippocampus and cerebellum (*P* < 0.01, *n* = 3–4 mice; **Figure 4c**,**d**). The upregulation of Ube3a was confirmed by western blot analysis of brain cytosolic lysates from three different mice that received TAT-S1 treatment (**Figure 4e**). In the western blot, the treatment-dependent appearance of a particular Ube3a isoform (the lower band) can be clearly seen. Both the immunohistochemistry and western blot confirm that the treatment increased Ube3a to a level intermediate between no-treatment AS and wild-type control mice. These results were replicated in an additional experiment in which the treatment was administered by i.p. injection for 7.5 weeks and visualized using a different antibody to Ube3a (**Supplementary Figure S3**).

## Discussion

Our study demonstrates that purified TAT-S1 protein can be injected s.c. or i.p. in AS mice, cross the blood–brain barrier, enter neurons throughout the brain, and alter the expression of Ube3a. We believe this to be the first report of an injectable engineered zinc finger repressor that can distribute widely in the brain and regulate the expression of an endogenous gene. Distribution of ATFs was observed by *in vivo* mCherry fluorescence throughout the body of the mouse, including the cranium. Widespread distribution in the brain was also confirmed by immunofluorescence based on a different part of the protein, the HA tag. Furthermore, we demonstrated that the full-length ATF is able to penetrate nuclei in cells of the brain (**Figure 2d**). TAT-S1 was able to significantly increase Ube3a expression in both the hippocampus and cerebellum, indicating that the portion of the protein necessary for *Ube3a-ATS* repression entered the nuclei of cells in the brain. While TAT-S1 must be nuclear to activate *Ube3a* expression, the Ube3a protein is not necessarily expected to be nuclear. The two anti-Ube3a antibodies used in the current study detected primarily the cytoplasmic form. Previous publications have noted that different isoforms of Ube3a localize to the nucleus, the cytoplasm or both,^24^ and roles have been proposed for Ube3a at synaptic junctions.^25^

The mechanism of TAT-mediated delivery of large proteins was reported to be adsorptive endocytosis.^26^ Although formally this study did not demonstrate that delivery was TAT-dependent, adsorptive endocytosis would likely allow the protein to enter many cell types, including both neurons and glia in the brain. However, the *Ube3a-ATS* is not expressed in glia, in these cells Ube3a is always expressed from both the maternal and paternal alleles. The fact that TAT-S1 upregulated Ube3a strongly suggests that the factors entered neurons, as this is where Ube3a is imprinted by the *Ube3a-ATS*, the target of TAT-S1. The observation that Ube3a expression was increased by TAT-S1 but not TAT-R6, which differed by only 26 aa in their DNA-binding domains, indicates that the function of TAT-S1 was dependent on the DNA-binding specificity of its zinc finger array. We note that the presence of the maltose-binding protein band in the injected material did not appear to influence the results, as both the TAT-S1 and TAT-R6 samples contained this band but only TAT-S1 increased Ube3a. Therefore, increased Ube3a expression was not caused by potential nonspecific factors such as stress or potential immune response in the mouse, contaminants in the injection solution, or nontargeted actions by other parts of the protein such as detachment of the KRAB domain.

In this study, S1 treatment only partially restored Ube3a expression to wild-type levels. This intermediate level was expected since previous therapeutic approaches using topoisomerase inhibitors, antisense oligonucleotides, and stop codon insertions had resulted in partial restoration of Ube3a from the paternal allele.^9,10,11^ TAT-R6 appeared to cause a slight, but not significant, decrease in Ube3a levels in the experiment shown in **Figure 3**, but a slight, but not significant, increase in the experiment shown in **Supplementary Figure S3**. These fluctuations are likely due to experimental variation using different cohorts on different days. In the current study, the partial reactivation by TAT-S1 may have been caused by an insufficient concentration of TAT-S1 at the target site. Future experiments could increase either the dose or the timing of administrations. The partial response might also be due to additional *Ube3a-ATS* transcripts initiating from upstream promoters.^8^ Future experiments could examine additional ATFs that would inhibit these promoters and perhaps be delivered as a combinational therapy.

The prospect for long-term treatment with proteins has been demonstrated by insulin and numerous other biologics.^27^ In principle, the use of ATF biologics with short-term effects similar to drugs may have advantages over approaches such as permanent gene engineering, for which distribution, dosage and reversibility are problematic.^12^ The Ube3a-ATS transcript is only expressed in mature neurons of the brain (reviewed in ref. 28); therefore, we do not expect that TAT-S1 will affect Ube3a expression in non-neuronal cells. No overt toxicity was observed over a 4-week treatment period in the current study. Longer-term studies might reveal innate or adaptive immune responses to the treatment. ATFs designed to alter epigenetic information may eventually provide more persistent gene regulatory effects,^29,30^ reducing costs and potential immune risks. Methods that restrict delivery to neurons only may help reduce amount of protein required. In addition, other programmable platforms such as TALE and CRISPR/Cas systems may be useful for gene regulation in the brain, even as purified protein.^31,32^ The development of an ATF approach could have significant impact beyond AS, since in other neurological disorders, only small adjustments to the DNA-binding domain of the ATF would be required to target disease-related loci that are imprinted (Rett, Prader–Willi), dominant (Huntington), or haploinsufficient (22q11.2 deletion, Pitt-Hopkins, and many autism spectrum disease genes).

## Materials and Methods

**In vitro *transcription factor assays.*** Zinc finger coding regions were designed by modular assembly methods^33^ and synthesized by BioBasic (Markham, Canada). The arrays were cloned using *Xho*I and *Not*I into the PGK promoter-driven mammalian expression vector pPGK-VP64,^34^ which appended an N-terminal HA epitope tag and SV40 nuclear localization sequence, and either a C-terminal VP64 transcriptional activation domain^18^ or a KRAB transcriptional repression domain, cloned at the *Not*I and *Pst*I sites.^18^ Recognition helices of the zinc finger arrays are provided in **Supplemental Table S1**. Target sites for the ATFs were cloned between *Not*I and *Xho*I sites upstream of the SV40 promoter in pGL3-control plasmids (Promega, Madison, WI), as listed in **Supplementary Figure S1**. In 24-well plates, HEK293T cells at 80% confluency in Dulbecco's modified Eagle's medium supplemented with 10% fetal calf serum, 1 U/ml of penicillin, and 1 µg/ml of streptomycin were cotransfected with 100 ng of ATF expression plasmid, 25 ng of modified pGL3-control firefly luciferase reporter plasmid containing ATF target sites, and 25 ng of pRL-TK-Renilla Luciferase plasmid (as a transfection control, Promega), using Lipofectamine 2000 (Invitrogen, Carlsbad, CA). Cells were harvested 48 hours posttransfection by removing media, washing with 500 µl of 1× Dulbecco's phosphate-buffered saline (Life Technologies, Carlsbad, CA), followed by lysis in 100 µl of 1× Passive Lysis Buffer (Promega) with 1× Complete Protease Inhibitors (Roche, Basel, Switzerland). Clarified cell lysates (20 µl) were used to determine luciferase activity using DualGlo reagents (40 µl, Promega) in a Veritas microplate luminometer (Turner Biosystems, Sunnyvale, CA). The firefly luciferase signal was normalized to renilla luciferase controls. All experiments were performed in duplicate and repeated on 3 different days. 

***Expression of TAT-S1 and TAT-R6 protein.*** TAT-ATF coding regions were cloned into the pMAL-c2X vector (New England Biolabs, Ipswich, MA) between *Eco*RI and *Hind*III. The full sequence of the TAT-S1 protein is provided in **Supplemental Figure S1**, and the recognition helices of TAT-R6 are provided in **Supplemental** **Table S1**. Proteins were expressed in NEB5α *E. coli* (New England Biolabs) by inoculating 5 ml overnight cultures into 800 ml of Luria Broth medium (Sigma, St Louis, MO) with 50 µg/ml Carbenicillin. Cultures were shaken overnight at 37 °C, then moved to 4 °C, and induced with isopropyl β-d-1-thiogalactopyranoside (0.5 mmol/l) and gently shaken for 48–72 hours. The cold temperature induction was critical to obtaining high yields; induction at room temperature or 37 °C was far less efficient. The culture was pelleted and resuspended in 30 ml of Zinc Buffer A (ZBA; 10 mmol/l Tris (pH 8.5), 90 mmol/l KCl, 1 mmol/l MgCl_2_, 100 µmol/l ZnCl_2_). The resuspended pellet was microfluidized, which was critical for obtaining full-length protein. Microfluidized lysates were run over columns of amylose resin (New England Biolabs, E8021L) that had been prepared by washing twice with deionized water, twice with Column Buffer (20 mmol/l Tris–HCl (pH 7.4), 0.2 mol/l NaCl, 1 mmol/l ethylenediaminetetraacetic acid (EDTA)) and twice with ZBA. Protein was eluted in Elution Buffer (ZBA, 500 mmol/l maltose) and concentrated to ~16 mg/ml using a Centricon Plus-70 (Millipore, Billerica, MA, UFC710008). Proteins were sterile filtered to remove any residual bacteria and routinely checked to assure no detectable endotoxins were present. Protein integrity was evaluated using Coomassie-stained gels and concentration established via Nanodrop A280 UV absorption. Protein concentrations for injections refer to the full-length + maltose-binding protein band intensities, of which only half was considered to be the 100-kD full-length protein (**Supplementary Figure S2**). Proteins were stored at −80 °C in ATF Injection Buffer (Elution Buffer, 30% glycerol, 4 mmol/l dithiothreitol). A dose of 160–200 mg/kg consisted of a 0.25 ml injection i.p. or s.c. containing 4 mg of total protein in a 20–25 kg mouse.

***Electromobility shift assays.*** Biotin-labeled DNA targets were generated by PCR-amplification using a 5′ biotinylated forward primer of 69-mer oligonucleotides containing 18-base pair zinc finger target site (**Supplementary Table S2**). PCR reactions contained unlabeled reverse primer in a 4:1 ratio over the biotinylated primer. Amplified targets were column purified (Qiagen, Hilden, Germany). Protein–DNA complexes were mixed on ice and then incubated in the dark for 1.5 hours at room temperature in ZBA, 0.05% NP-40, 0.1 mg/ml bovine serum albumin, 10% glycerol, and 35–75 pmol/l target oligo. Protein–DNA complexes were separated from the unbound probe using 7% polyacrylamide Tris/boric acid/EDTA gels run in 0.5× Tris/boric acid/EDTA (BioRad, Hercules, CA) and then transferred onto Biodyne B nylon membranes. Complexes were UV cross-linked to the membranes for 4 minutes (UV Stratalinker 1800, Agilent, Santa Clara, CA). The biotinylated DNA was visualized using the LightShift Chemiluminescent EMSA Kit (Pierce, Waltham, MA) according to the manufacturer's protocol. Equilibrium binding constants (apparent *K*_D_) were calculated from protein titration experiments. Gel images on X-ray film (Denville Scientific, South Plainfield, NJ) were scanned and then quantitated using ImageJ. All reported EMSA measurements were averages of at least three experiments performed with independent protein dilutions. Representative data are shown in **Figure 1b**.

***Chromatin immunoprecipitation assay.*** The mouse neuroblastoma cell line Neuro2a (ATCC #CCL-131) was grown in Dulbecco's modified Eagle's medium supplemented with 10% bovine calf serum. Neuro2a cells were grown to 70% confluency and transfected with 15 µg S1-KRAB-expressing plasmid per 10-cm dish using Lipofectamine 2000 (Life Technologies). Cells were cross-linked 48 hours posttransfection by incubation with 1% formaldehyde solution for 10 minutes at room temperature. Chromatin immunoprecipitation assays were performed as previously described with minor modifications.^35^ About 30 µg of sonicated chromatin was incubated with 3 µg of monoclonal anti-HA antibody (Covance, San Diego, CA, HA.11 clone 16B12), monoclonal anti-RNA Polymerase II antibody (Covance 8WG16), or control mouse IgG (Sigma). Immunoprecipitates were captured using 3 µg rabbit anti-mouse serum and StaphA cells (Sigma). After washes and reversal of DNA–RNA–protein cross-links, standard and quantitative PCR were performed to confirm specific binding to the S1 target sequence in chromatin immunoprecipitation samples as compared to 0.1% input control or relative to binding at an unrelated promoter on chromosome 4. All primer sequences are listed in **Supplementary Table S2**. Primers Snurf-F and Snurf-R to the mouse *Snurf* gene promoter target site were used as positive control primers, while mmchr4-F and mmchr4-R to a region on mouse chromosome 4 served as a negative control. Specific fragments were amplified using GoTaq (Promega) DNA polymerase (2 minutes at 95 °C; 30 seconds at 95 °C, 30 seconds at 60 °C, 30 seconds at 72 °C, 35 cycles; 5 minutes at 72 °C). PCR products were separated on a 1.5% agarose gel and visualized using the Gel Doc XR+ System (BioRad). Quantitative real-time PCR was used to calculate binding enrichment using the ΔΔ*C*_t_ method.

***Bind-n-Seq assay.*** Bind-n-Seq was performed essentially as previously described.^21^ Briefly, we used barcoded 93-mer double-stranded oligonucleotide targets containing Illumina (San Diego, CA) primer binding sites and a 21-nt random region. Various protein concentrations were incubated with 3 µmol/l oligo target, 0.12 µg/µl herring sperm DNA, 100 µmol/l ZnCl_2_, 5 mmol/l dithiothreitol, 1% bovine serum albumin, 5% glycerol, and 1, 50, or 100 mmol/l KCl. Bound complexes were precipitated using amylose resin and then washed with a corresponding buffer of 10 mmol/l Tris pH 8.5, 100 µmol/l ZnCl_2_, 1 mmol/l MgCl_2_, 5 mmol/l dithiothreitol, and 1, 50, or 100 mmol/l KCl. Eluted and amplified DNA was sequenced on an Illumina HiSeq2000 or MiSeq. Motifs were determined using MERMADE, available at korflab.ucdavis.edu/Datasets/BindNSeq/.

***Mice.*** All mice were maintained and experiments conducted according to University of California, Davis approved Institutional Animal Care and Use Committee (IACUC) guidelines and protocols. The AS mouse model^16^ was obtained from Jackson Laboratories (Bar Harbor, ME, Stock 004477, 129-*Ube3a*^*tm1Alb*^/J) and maintained on a mixed background of SV129 and C57BL/6. Wild-type C57BL/6 mice (Jackson Laboratories) and SV129 mice (Charles River Laboratories, Wilmington, MA) were used for matings with *Ube3a*-deficient female mice. Genotyping was performed according to the supplier's protocol, with the modification that the annealing temperature was changed to 60 °C. Primers are listed in **Supplementary Table S2**. All animals receiving injections were older than 2 months of age at the time of the first injection.

***Maestro* in vivo *imaging of mice.*** All mice used in the initial blood–brain barrier study were C57BL/6 male mice. All mouse images were performed on a Maestro 2 Imager (PerkinElmer, Waltham, MA), use and training provided by the Center for Molecular and Genomic Imaging at UC Davis. The green filter was used with acquisition settings of 550–800 nm in 10-nm steps. Before injections, an image was taken of the purified protein using the auto-expose option. Timepoints used were 15 minutes, 4 hours, 8 hours, and 24 hours postinjection. At each timepoint, fluorescent images were taken of the injection site, the head and the back. Exposure times for the injection site, head, and back images were established from a previous pilot experiment at 229, 1,959, and 1,986 ms, respectively. On all images, a mock-treated animal served as a control, mock was the ATF/protein elution buffer of ZBA, 500 mmol/l maltose. All images were based on the same raw inputs for mCherry signal and background mouse signal. Analysis of mCherry signal was performed by measuring the mCherry by using a consistent sized area in the brain region using Maestro software. The measurement area was the same for each mouse across all treatments. Additional later images were taken of the *Ube3a*-deficient mice when treated, to ensure brain localization of the TAT-ATFs.

***Immunohistochemistry.*** For the images in **Figure 4b**,**c** (50-µm-thick sections), one brain hemisphere was washed in 1× TBS (50 mmol/l Tris–Cl, pH 7.5, 150 mmol/l NaCl), fixed in 10% buffered paraformaldehyde overnight, and then placed into 30% sucrose for 3 days. Following brain saturation, the tissue was frozen in Tissue-Tek CRYO-OCT Compound (Thermo-Fisher, Waltham, MA, 14-373-65) and then sectioned on a Leica (Nussloch, Germany) cryotome. Blocking was performed using Superblock (Life Technologies) for 1 hour, followed by a 10% goat serum, 0.3% Triton X in 1× TBS for 2 hours. Following aspiration, slides were stained with primary anti-HA 1:150 (Roche 12013819001) that was directly labeled with an Apex Antibody labeling kit (Life Technologies, A10470), or anti-Ube3a 1:100 (Sigma E8655) labeled with the Apex Antibody labeling kit, in 5% goat serum and 0.15% Triton X and incubated at 4 °C overnight. Slides were washed three times with 1× TBS, stained with 4′,6-diamidino-2-phenylindole for 10 minutes, washed three times with 1× TBS, and then mounted with coverslip using Prolong Gold (Life Technologies). Images were acquired using a Leica DM6000B epifluorescent microscope. A 10% linear reduction in brightness was applied equally to the green channel of all images to reduce autofluorescence and clarify features. All fluorescence intensity measurements were performed on unaltered images using ImageJ software 1.48v. Fluorescence intensities were measured in the hilus region of the dentate gyrus in the hippocampus and the Purkinje cell layer in the cerebellum.

For the images in **Supplementary Figure S3** (5-µm sections), paraffin embedding was utilized. One hemisphere of the brain was placed in 1× TBS and then fixed in 4% paraformaldehyde overnight. Brains were fixed in an additional 3 hours in 10% paraformaldehyde solution and then placed into wax for sectioning. Slides of these sectioned tissues were incubated overnight at 56 °C, washed with xylene twice for 10 minutes, and then soaked in 100% ethanol twice for 10 minutes. Antigen retrieval was performed using 1× Target Retrieval Solution (Dako, Carpinteria, CA) at 95 °C for 1 hour and then washed in 1× SSC (150 mmol/l NaCl, 15 mmol/l Sodium Citrate) at room temperature for 5 minutes while rocking gently. Blocking was performed using Superblock for 1 hour, followed by a 10% goat serum, and 0.3% Triton X in 1× TBS for 2 hours. Following aspiration, slides were stained with primary anti-Ube3a 1:1,000 (Atlas, Stockholm, Sweden, HPA039410) with coverslip and incubated at 4 °C overnight. Coverslip were removed and the slide was washed three times with 1× TBS. The secondary antibody, goat-anti-rat Alexa Fluor 647 1:1,000 (Abcam, Cambridge, MA, 150159) or goat-anti-rabbit Alexa Fluor 488 1:1,000 (Abcam 150077) in 1× TBS, 5% goat serum, and 0.15% Triton, was applied for 2 hours, the slides were then washed three times with 1× TBS, and then dried before placing coverslips. Coverslips were applied with 4′,6-diamidino-2-phenylindole-containing Vectashield (Vector Laboratories, Burlingame, CA, H-1200). The images in **Supplementary Figure S3** received no alterations. All fluorescence intensity measurements were performed on unaltered images as above.

***Western blot.*** For the experiments in **Figure 2d**, one hemisphere from each brain was flash frozen in liquid nitrogen. Tissues were treated first with hypotonic buffer (2 mmol/l 4-(2-hydroxyethyl)-1-piperazineethanesulfonic acid (HEPES) (pH 5), 1 mmol/l KCl, 0.1 mmol/l EDTA, 10% Glycerol, 1× protease inhibitor cocktail (Roche, 11873580001), 50 µmol/l phenylmethylsulfonyl fluoride, 50 µl of 100 µmol/l dithiothreitol, in diethylpyrocarbonate-treated water) and then ground with motorized pestle for 35 seconds. The remaining nuclear pellet after centrifugation was lysed with radioimmunoprecipitation assay buffer (50 mmol/l Tris–Cl, pH 7.4, 150 mmol/l NaCl, 0.25% NaDeoxycholate, 1% NP-40 (IGEPAL), 1 mmol/l EDTA, 1× Protease Inhibitor Cocktail). The nuclear lysate was quantified using a BCA assay (Life Technologies). Polyacrylamide gels (10–20% Tris Glycine, BioRad) were loaded with 250 µg of protein per sample and run at 115 V for 1.5 hours and then transferred to polyvinylidene difluoride membrane using a semi-dry blotting apparatus at 75 V for 1.5 hours. The membrane was blocked in 5% dry milk in TBST (20 mmol/l Tris, pH 7.5, 150 mmol/l NaCl, 0.1% Tween) and then probed for 48 hours at 4 °C with primary a rat anti-HA antibody conjugated to horseradish peroxidase at 1:100–250 (Roche, 12013819001). The western was incubated with the ECL Plus Reagent (Amersham, Marlborough, MA, RPN2133) for 15 minutes and imaged on a Storm 860 imager (Molecular Dynamics, Marlborough, MA). Images were not altered.

For the experiments in **Figure 4e**, mouse brains were harvested and immediately snap frozen at −80 °C. Proteins were extracted using the CelLytic NuCLEAR Extraction Kit (Sigma) according to the manufacturer's instructions. Protein concentration was determined utilizing the BCA Protein Assay kit (Pierce). About 20 µg of protein were separated on a Novex 4–12% Bis-Tris gel (Life Technologies) using 3-(*N*-morpholino)propanesulfonic acid buffer and BOLT Antioxidant as described by the manufacturer. Using the Mini Trans-Blot Cell (BioRad), proteins were transferred onto nitrocellulose membranes, in transfer buffer (25 mmol/l Tris, 192 mmol/l glycine, 20% (v/v) methanol), overnight at 30 V in the cold room. After transfer, protein loading was evaluated using Ponceau S staining. Membranes were then blocked with 5% dry milk in TBST (20 mmol/l Tris, pH 7.5, 150 mmol/l NaCl, 0.1% Tween) and probed with anti-Ube3a antibody (Sigma, E8655, 1:1,000 dilution). After incubation with goat anti-mouse horseradish peroxidase (Santa Cruz, Biotechnology, Santa Cruz, CA, SC2055, 1:2,500 dilution), chemiluminescence was detected using the ECL Prime Western Blotting Detection Reagent (Amersham, RPN2232).

**SUPPLEMENTARY MATERIAL** **Figure S1.** Sequence of proteins and reporters used in this study. **Figure S2.** TAT-ATFs protein purification. **Figure S3.** High-resolution images of Ube3a activation by TAT-S1 but not TAT-R6. **Table S1.** DNA binding sequence and recognition helices of the zinc finger arrays used in this study. **Table S2.** Primers used in this study.
