# Supplementary material for: Protein Delivery of an Artificial Transcription Factor Restores Widespread Ube3a Expression in an Angelman Syndrome Mouse Brain
Source: Mol Ther. 2016 Feb 2;24(3):548–55. doi: 10.1038/mt.2015.236 (PMC4786922; doi:10.1038/mt.2015.236)
Supplement: Supplementary Figures and Tables [file mt2015236x1.pdf]

SUPPLEMENTARY MATERIALS

Protein Delivery of Artificial Transcription Factor Restores Widespread Ube3a Expression in an Angelman Syndrome Mouse Brain

Barbara J. Bailus[1], Benjamin Pyles[1], Michelle M. McAlister[1], Henriette O'Geen[1], Sarah H. Lockwood[1], Alexa N. Adams[1], Jennifer T. Trang Nguyen[1], Abigail Yu, Robert F. Berman[2], and David J. Segal[1]*

[1]Genome Center, MIND Institute, and Department of Biochemistry and Molecular Medicine, University of California, Davis, CA 95616, USA
[2]Department of Neurological Surgery, University of California, Davis, CA 95616, USA

* To whom correspondence should be addressed:
djsegal@ucdavis.edu

TABLE OF CONTENTS

Figure S1: Sequence of proteins and reporters used in this study……………………..………. 2-4
Figure S2: TAT-ATFs protein purification………………..……………………………………… 5
Figure S3: High-resolution images of Ube3a activation by TAT-S1 but not TAT-R6……....... 6
Table S1: DNA binding sequence and recognition helices of the zinc finger arrays used in this study……………………………………………………………………………………......... 7
Table S2: Primers used in this study…………………………………………………………… 8

Figure S1: Sequence of proteins and reporters used in this study.

A) Sequence of the TAT-S1 ATF (922 aa)

Maltose binding protein-TAT-mCherry-HA-tag-SV40-NLS-**Zinc fingers (recognition helices F1 -> F6 underlined)**-KRAB-A box

Protein

MKIEEGKLVIWINGDKGYNGLAEVGKKFEKDTGIKVTVEHPDKLEEKFPQVAATGDGPDIIFW
AHDRFGGYAQSGLLAEITPDKAFQDKLYPFTWDAVRYNGKLIAYPIAVEALSLIYNKDLLPNP
PKTWEEIPALDKELKAKGKSALMFNLQEPYFTWPLIAADGGYAFKYENGKYDIKDVGVDNAGA
KAGLTFLVDLIKNKHMNADTDYSIAEAAFNKGETAMTINGPWAWSNIDTSKVNYGVTVLPTFK
GQPSKPFVGVLSAGINAASPNKELAKEFLENYLLTDEGLEAVNKDKPLGAVALKSYEEELAKD
PRIAATMENAQKGEIMPNIPQMSAFWYAVRTAVINAASGRQTVDEALKDAQTNSSSNNNNNNN
NNNLGIEGRISEFGSGAPGRKKRRQRRRVDMVSKGEEDNMAIIKEFMRFKVHMEGSVNGHEFE
IEGEGEGRPYEGTQTAKLKVTKGGPLPFAWDILSPQFMYGSKAYVKHPADIPDYLKLSFPEGF
KWERVMNFEDGGVVTVTQDSSLQDGEFIYKVKLRGTNFPSDGPVMQKKTMGWEASSERMYPED
GALKGEIKQRLKLKDGGHYDAEVKTTYKAKKPVQLPGAYNVNIKLDITSHNEDYTIVEQYERA
EGRHSTGGMDELYKSRYPYDVPDYANSPGIPGMGPKKKRKVGRLE**PGEKPYMCAECGKSFSRS**
DDLVRHQRTHTGEKPYKCPECGKSFSDCRDLARHQRTHTGEKPYKCPECGKSFSQRAHLERHQ
RTHTGEKPYKCPECGKSFSREDNLHTHQRTHTGEKPYKCPECGKSFSRSDDLVRHQRTHTGEK
PYKCPECGKSFSTSGNLTEHQRTHTGAAATLVTFKDVFVDFTREEWKLLDTAQQIVYRNVMLE
KYKNLVSLGYQLTKPDVILRLEKGEEPWLVEREIHQETHP*

DNA

ATGAAAATCGAAGAAGGTAAACTGGTAATCTGGATTAACGGCGATAAAGGCTATAACGGTCTCGCTGAAG
TCGGTAAGAAATTCGAGAAAGATACCGGAATTAAAGTCACCGTTGAGCATCCGGATAAACTGGAAGAGAA
ATTCCCACAGGTTGCGGCAACTGGCGATGGCCCTGACATTATCTTCTGGGCACACGACCGCTTTGGTGGC
TACGCTCAATCTGGCCTGTTGGCTGAAATCACCCCGGACAAAGCGTTCCAGGACAAGCTGTATCCGTTTA
CCTGGGATGCCGTACGTTACAACGGCAAGCTGATTGCTTACCCGATCGCTGTTGAAGCGTTATCGCTGAT
TTATAACAAAGATCTGCTGCCGAACCCGCCAAAAACCTGGGAAGAGATCCCGGCGCTGGATAAAGAACTG
AAAGCGAAAGGTAAGAGCGCGCTGATGTTCAACCTGCAAGAACCGTACTTCACCTGGCCGCTGATTGCTG
CTGACGGGGGTTATGCGTTCAAGTATGAAAACGGCAAGTACGACATTAAAGACGTGGGCGTGGATAACGC
TGGCGCGAAAGCGGGTCTGACCTTCCTGGTTGACCTGATTAAAAACAAACACATGAATGCAGACACCGAT
TACTCCATCGCAGAAGCTGCCTTTAATAAAGGCGAAACAGCGATGACCATCAACGGCCCGTGGGCATGGT
CCAACATCGACACCAGCAAAGTGAATTATGGTGTAACGGTACTGCCGACCTTCAAGGGTCAACCATCCAA
ACCGTTCGTTGGCGTGCTGAGCGCAGGTATTAACGCCGCCAGTCCGAACAAAGAGCTGGCAAAAGAGTTC
CTCGAAAACTATCTGCTGACTGATGAAGGTCTGGAAGCGGTTAATAAAGACAAACCGCTGGGTGCCGTAG
CGCTGAAGTCTTACGAGGAAGAGTTGGCGAAAGATCCACGTATTGCCGCCACCATGGAAAACGCCCAGAA
AGGTGAAATCATGCCGAACATCCCGCAGATGTCCGCTTTCTGGTATGCCGTGCGTACTGCGGTGATCAAC
GCCGCCAGCGGTCGTCAGACTGTCGATGAAGCCCTGAAAGACGCGCAGACTAATTCGAGCTCGAACAACA
ACAACAATAACAATAACAACAACCTCGGGATCGAGGGAAGGATTTCAGAATTCGGATCTGGCGCGCCAGG
CCGTAAAAAACGTCGTCAGCGCCGTCGCGTCGACATGGTGAGCAAGGGCGAGGAGGATAACATGGCCATC
ATCAAGGAGTTCATGCGCTTCAAGGTGCACATGGAGGGCTCCGTGAACGGCCACGAGTTCGAGATCGAGG
GCGAGGGCGAGGGCCGCCCCTACGAGGGCACCCAGACCGCCAAGCTGAAGGTGACCAAGGGTGGCCCCCT
GCCCTTCGCCTGGGACATCCTGTCCCCTCAGTTCATGTACGGCTCCAAGGCCTACGTGAAGCACCCCGCC
GACATCCCCGACTACTTGAAGCTGTCCTTCCCCGAGGGCTTCAAGTGGGAGCGCGTGATGAACTTCGAGG
ACGGCGGCGTGGTGACCGTGACCCAGGACTCCTCCCTGCAGGACGGCGAGTTCATCTACAAGGTGAAGCT
GCGCGGCACCAACTTCCCCTCCGACGGCCCCGTAATGCAGAAGAAGACCATGGGCTGGGAGGCCTCCTCC
GAGCGGATGTACCCCGAGGACGGCGCCCTGAAGGGCGAGATCAAGCAGAGGCTGAAGCTGAAGGACGGCG
GCCACTACGACGCTGAGGTCAAGACCACCTACAAGGCCAAGAAGCCCGTGCAGCTGCCCGGCGCCTACAA

CGTCAACATCAAGTTGGACATCACCTCCCACAACGAGGACTACACCATCGTGGAACAGTACGAACGCGCC
GAGGGCCGCCACTCCACCGGCGGCATGGACGAGCTGTACAAGTCTAGATACCCATACGATGTCCCAGACT
ACGCGAATTCCCCGGGGATCCCAGGCATGGGGCCCAAAAAGAAACGCAAAGTTGGGCGCCTCGAG**CCCGG**
GGAGAAACCATATAAGTGTCCAGAGTGTGGTAAATCCTTTAGCAGAAGCGATGATCTGGTGAGACATCAA
AGAACACACACCGGAGAAAAGCCTTACAAGTGTCCTGAGTGCGGTAAGAGCTTTTCTGATTGTAGAGACC
TTGCGCGCCATCAGCGGACTCACACAGGCGAGAAGCCTTATAAATGCCCTGAGTGTGGCAAATCTTTCTC
TCAGAGAGCACATCTGGAGCGACATCAGAGGACGCACACTGGGGAGAAACCCTATAAGTGCCCTGAATGC
GGAAAAAGTTTTAGTCGGGAAGATAACTTGCATACACACCAGAGGACACATACGGGCGAAAAGCCCTACA
AATGTCCGGAATGCGGGAAGTCCTTCTCTCGGTCAGATGACCTCGTTAGGCACCAGAGAACACATACCGG
CGAAAAACCTTACAAATGCCCAGAATGTGGGAAGAGCTTCAGCACTTCCGGCAATCTTACCGAGCACCAA
AGAACCCACACCGGTGCGGCCGCCACACTGGTGACCTTCAAGGATGTATTTGTGGACTTCACCAGGGAGG
AGTGGAAGCTGCTGGACACTGCTCAGCAGATCGTGTACAGAAATGTGATGCTGGAGAAATATAAGAACCT
GGTTTCCTTGGGTTATCAGCTTACTAAGCCAGATGTGATCCTCCGGTTGGAGAAGGGAGAAGAGCCCTGG
CTGGTGGAGAGAGAAATTCACCAAGAGACCCATCCTTAGCTGCAGAAGCTT

B) SR-KRAB and AT-KRAB ATF luciferase reporter:
This segment was inserted NheI/XhoI into pGL3-ctrl (Promega).
GCTAGCAGAGTGTGAGCACTGGGCAAAGTTGCCCACCCTGGAGGGCGCGGAGGGACTGGTGGGC
TGGGACGGGGAGGAGCCGGCCAAGAGGGCGGGATGGAATAGGAAAATACAGGTGAGGGGGAGGG
TGTTGTAAAGGGGGAGCAGGAAAAGGAGGGTACAGAAGCAGGGGTCAAAGTGGGAGCTCCTAGG
GTGGATGTGACAGCAGGACCTGCTGCACTGGGGGTTGGGAGTGTGATTCTCGAG

C) SNF-KRAB ATF luciferase reporter:
This segment was inserted NheI/XhoI into pGL3-ctrl (Promega).
S1 site is underlined.
GCTAGCTCCATTGCGTTGCAAATCACTCCTCAGAACCAAGCGTCTGGCATCTCCGGCTCCCTCT
CCTCTCTGCGCTAGTCTTGCCGCAATGGCTCAGGTTTGTCGCGCGGCTCCCTACGCATGCGTCC
CAGGCAATGGCTGCACATGCGCACATTTTTGCCGCAATGCAGGGGTCTCTGTCCCTCTGACCGG
AATGTCCTGCCAAAAGGCAGCTACCAAAAGGAATGCTTGAGCATTCCTACTGCGGAAATTTGAG
CGTGTATTAAAAAAAAAAAAAATCACTGTCAATCTGGAATAATGGATATAATTGCCTATTACTAA
ATACATTTAATTTTTTAAAAAAATTGCTCCCAAGTGATTGCAAGCATCACACAGATTTTTATTA
TGTGGGAGAGGAAAAAAGGTTTGATCTCCAATATGAGGAAGACTGGTTTCTAGGCTATATCATG
TTACATAGTGTATCACACACACACACACACACACACACACACACACAATCAAACATGAAGCCCA
GCCAAATTGTCAGATACTTAAAATTCTGTACAGAAAAAAAAGTTATCCTGATTCCTAATTCTAC
ATTCTACATTAATGTAGTATTACTTGTATTTTCTTTCTCTGTGTTAGAACATATGACTGGGAGA
AAACTATTTCCTCCAGATCATGGTGTGATTAAATTTTAATAGGAAGAACATTCAGTTATGAAAC
TTATGTATCTGCTTCTTTTGTTGTTGTTGGTTTTAGAGACAGGGCTTCCTAAATGTTGTTATGC
CTGGGGGGTGAAAAGTAGACACTATTTACTAACAAATATATGACCTCATAGAAATGCCATCCAG
TAATTCCAGCTCTTAGGAAACTTATTCTAAGAAAGCAGTATTTTTGCATACTCAAGGCCAGGTT
CAATTTGTAACAATATCTAAAAACAAAAACAAAAACATTTTGTAGATTGTGGATACATATATAA
GAATTTATTTCAGTTACTTTTGACCTATTAATTTCCTGTCTGCCTACTCGAG

D) Ube3a-VP64 ATF luciferase reporter:
This segment was inserted XhoI/HindIII into pGL3-ctrl (Promega), replacing the SV40 promoter.
CTCGAGGTGGGTTCCAGGACAGCCAGGGCTTCACAGAGAAATCTGTCTTGAAAAACCAATAATA
AAAATATTTTTTATAGTGGGGAGATCTTTCATATATATATGATGTGTATATATATATACACACA
CATACACACACATTATAGGAGCTGTAATAAACAGAAAAGCACATTAATACATGTGATTGTTACA
TTTTGAGCACTAGATGATTAAGGCTTACTAAGTTGCAAGCCAGGAAATAACATGTCAGCTAGTC
TTTATAATAATTTAGTGTCAAAATCATTAACAACAAACCTAAGTTAAACCCTCACTCCGGTACC

AACAAGTCACATGGCTCTAACAGGGTTCGGGTGTTCTGTAAATCAAAATTAGTCTCTATCCACG
TGTGATCAAGCAAGGCGGTTTCTTCTCAGTTGACATCTACTGGAAGACAACACAGGAGGGGCTG
GGCTTTTACCTACTAGTCTGGTAAGCCAGTGACATATCATGTTCTGGCAGAGGCTGATGGCCTT
GTTTGCTGAAAGGTTGCCCAATCTCAACGCCTGAATCCTTTACTCGGACAGTTCGGATGAAAAG
TCGTAAGGGTCAGCTCTAGCAACAAAACGCTTCTATGCCTCCTCGTGCCGCCAGTAGGCTACAC
CCTCTTGGCCTATTCGTCCCTCTGACTCCTGGGCTTTGCTAGGCTGGGTAGACCTGTCCGCTCT
CGCGAGTACACCGAAGCACACTGGTCTATAGATTGAGTAAAAGGAGCAGAGGCTAAAAACACCA
AGCCTCTCTCAGCCCAGTCTCAAGATGGCGACGATGCTGGCCAGCTTCCACCCAGCCCAAGGCC
ATCTCCCCTTCCCAAAGGCCCCTGCGCCGCAGGCGAAGGCGAGCTAGAGGAGGGCCCCACAGAG
CTGCGATCGGGGCGGGGCAGAGCCCGTCGCCATCCCACGCACGCGCAACCCCGCCTCGCATCCC
GCCTCCGGACTCCGAGCCCTCGCCCGGCAGGGTTGGCGCGCGCTGCAAGCTT

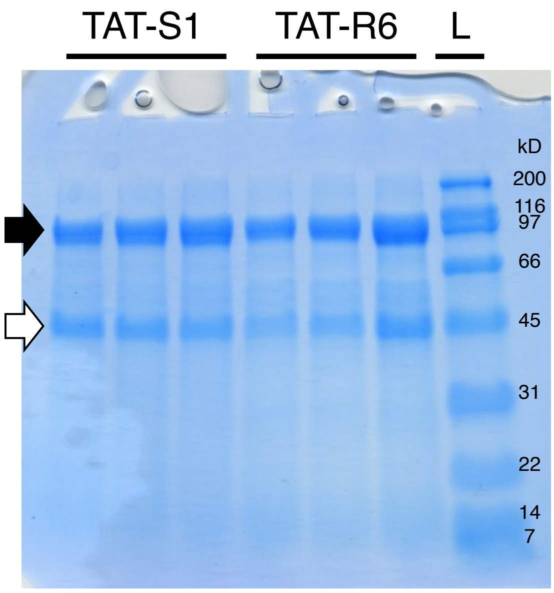

Figure S2: TAT-ATFs protein purification. Coomassie-stained gel demonstrating the purity of three separate preparations of TAT-S1 and TAT-R6 protein. Filled arrow, 100 kD full-length protein band is actually two close bands representing proteins with and without an attached MBP domain. Open arrow, 44 kD maltose binding protein.

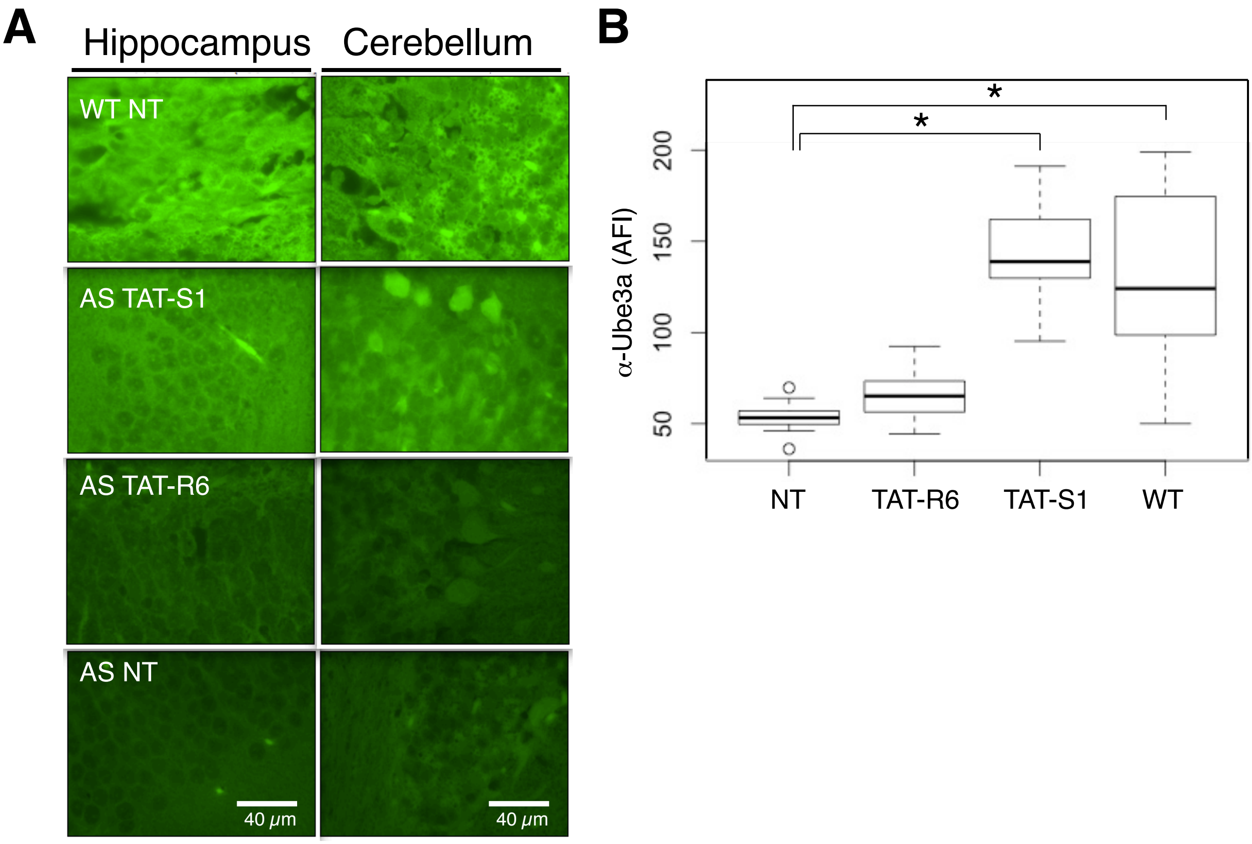

Figure S3: High resolution images of Ube3a activation by TAT-S1 but not TAT-R6. A mouse model of Angelman Syndrome (AS) was injected with TAT-S1 or TAT-R6 (160–200 mg/kg, intraperitoneal [IP]) three times per week for 7.5 weeks. **A**) High-resolution imaging of Ube3a protein expression in brain slices of the hippocampus hilus region of the dentate gyrus and the cerebellum Purkinje cell layer (α-Ube3a [Atlas HPA039410], 5 μm). Sections from no treatment (NT) wild type and AS mice are shown as controls. All images are unaltered, with no adjustment for autofloresence. **B**) Quantification of Ube3a from unaltered images of the same regions in different mice. One-way ANOVA found significant difference between groups [$F(3,22)=16.7$, $p<0.0001$], n = 3-4 mice. *, $p < 0.001$, post-hoc Tukey-Kramer HSD; AFI, average fluorescence intensity.

Table S1: DNA binding sequence (5' -> 3') and recognition helices (positions -1, 1, 2, 3, 4, 5, & 6 for fingers F6 -> F1) of the zinc finger arrays used in this study.

ZF Name	F6	F5	F4	F3	F2	F1
**A4**	CCA	GAC	TAG	TAG	GTA	AAA
helices	TSHSLTE	DPGNLVR	REDNLHT	REDNLHT	QSSSLVR	QRANLRA
**A6**	GGC	CAA	GAG	GGT	GTA	GCC
helices	DPGHLVR	QSGNLTE	RSDNLVR	TSGHLVR	QSSSLVR	DCRDLAR
**A7**	ATA	GAT	TGA	GTA	AAA	GGA
helices	QKSSLIA	TSGNLVR	QAGHLAS	QSSSLVR	QRANLRA	QRAHLER
**A8**	GCC	GCA	GGC	GAA	GGC	GAG
helices	DCRDLAR	QSGDLRR	DPGHLVR	QSSNLVR	DPGHLVR	RSDNLVR
**A9**	AGG	GCT	CGG	AGT	CCG	GAG
helices	RSDHLTN	TSGELVR	RSDKLTE	HRTTLTN	RNDTLTE	RSDNLVR
**AT56**	GGT	GAG	GGG	GAG	GGT	GTT
helices	TSGHLVR	RSDNLVR	RSDKLVR	RSDNLVR	TSGHLVR	TSGSLVR
**AT74**	ATG	GAA	TAG	GAA	AAT	ACA
helices	RRDELNV	QSSNLVR	REDNLHT	QSSNLVR	TTGNLTV	SPADLTR
**S1**	CAT	GCG	TAG	GGA	GCC	GCG
helices	TSGNLTE	RSDDLVR	REDNLHT	QRAHLER	DCRDLAR	RSDDLVR
**S2**	GCA	ATG	GCT	GCA	CAT	GCG
helices	QSGDLRR	RRDELNV	TSGELVR	QSGDLRR	TSGNLTE	RSDDLVR
**S3**	GAT	CTG	GAG	GAA	ATA	GTT
helices	TSGNLVR	RNDALTE	RSDNLVR	QSSNLVR	QKSSLIA	TSGSLVR
**SR71**	GCA	GGA	CCT	GCT	GCA	CTG
helices	QSGDLRR	QRAHLER	TKNSLTE	TSGELVR	QSGDLRR	RNDALTE
**SR115**	CCT	AGG	GTG	GAT	GTG	ACA
helices	TKNSLTE	RSDHLTN	RSDELVR	TSGNLVR	RSDELVR	SPADLTR
**R6 (neg ctrl)**	AAA	GTT	GCC	CAC	CCT	GGA
helices	QRANLRA	TSGSLVR	DCRDLAR	SKKALTE	TKNSLTE	QRAHLER

Table S2: Primers used in this study

Name	Sequence
	Primers for ChIP-PCR
Snurf-F	5’-CTCTCCTCTCTGCGCTAGTC-3’
Snurf-R	5’-AGAGACCCCTGCATTGCG-3’
mmchr4-F	5’-GAGCTATGGCCCATTGATGT-3’
mmchr4-R	5’-AATAGTGGGATGGTGGGAGA-3’
	Sequencing primers for Luciferase plasmids
RVprimer3-f	5'CTAGCAAATAGGCTGTCC3'
GLprimer2-r	5'CTTTATGTTTTTGGCGTCTTCC3'
	EMSA of purified ATFs
Biotinylated f	/5Biosg/CCTCTTCGCTATTACGCCAGC
Primer r	5'CACCCTGACTCGAGTACGATCGAACGTTC
S1 target site	5'-CCTCTTCGCTATTACGCCAGC CATGCGTAGGGAGCCGCG GAACGTTCGATCGTACTCGAGTCAGGGTG-3'
	AS mice genotyping primers
R1965	5'GCTCAAGGTTGTATGCCTTGGTGCT3'
WTF1966	5'AGTTCTCAAGGTAAGCTGAGCTTGC3'
ASF1967	5'TGCATCGCATTGTCTGAGTAGGTGTC3'
